# The potential role of CDC20 in tumorigenesis, cancer progression and therapy: A narrative review

**DOI:** 10.1097/MD.0000000000035038

**Published:** 2023-09-08

**Authors:** Feng Xian, Caixia Zhao, Chun Huang, Jun Bie, Guohui Xu

**Affiliations:** a School of Medicine, University of Electronic Science and Technology of China, Chengdu, China; b Department of Oncology, Nanchong Central Hospital, The Second Clinical College of North Sichuan Medical College, Nanchong, China; c Department of Interventional Radiology, Sichuan Clinical Research Center for Cancer, Sichuan Cancer Hospital & Institute, Sichuan Cancer Center, Affiliated Cancer Hospital of University of Electronic Science and Technology of China, Chengdu, China.

**Keywords:** apoptosis, cancer progression, CDC20, CDC20-target inhibitors, tumorigenesis

## Abstract

The cell division cycle 20 homologue (CDC20) is known to regulate the cell cycle. Many studies have suggested that dysregulation of CDC20 is associated with various pathological processes in malignant solid tumors, including tumorigenesis, progression, chemoradiotherapy resistance, and poor prognosis, providing a biomarker for cancer diagnosis and prognosis. Some researchers have demonstrated that CDC20 also regulates apoptosis, immune microenvironment, and tumor angiogenesis. In this review, we have systematically summarized the biological functions of CDC20 in solid cancers. Furthermore, we briefly synthesized multiple medicines that inhibited CDC20. We anticipate that CDC20 will be a promising and effective biomarker and therapeutic target for the treatment of human cancer.

## 1. Introduction

**Figure 1.**
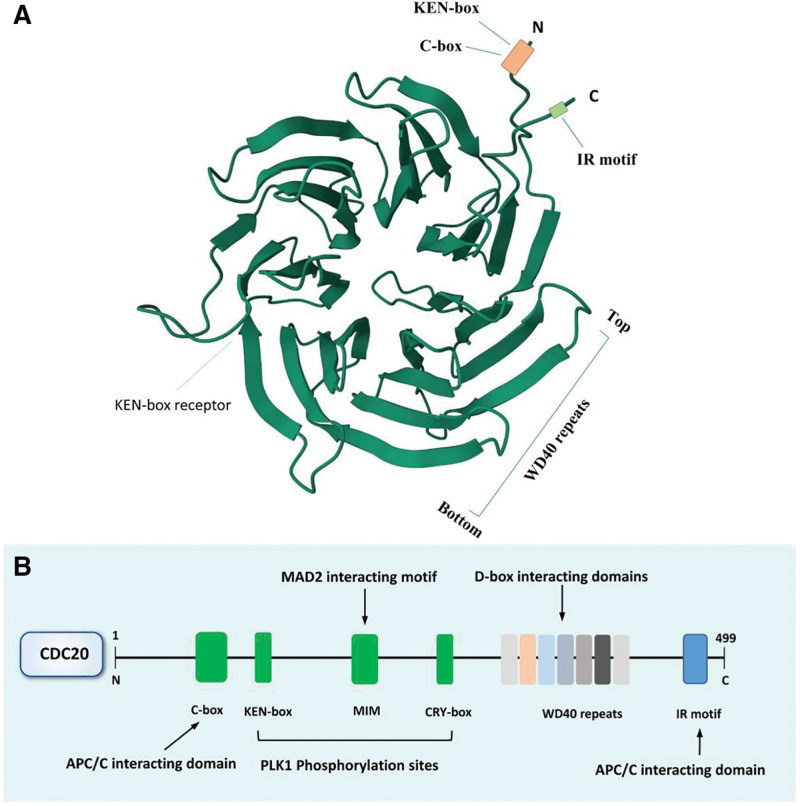
Cell division cycle 20 homologue (CDC20) domains and motifs. (A) 3D structure of CDC20 (http://www.rcsb.org). (B) Structure of human CDC20 with its KEN box, C-box, MAD2-interacting motif (MIM), 7 WD40 repeats, CRY box and IR motif.

The cell cycle of eukaryotic cells consists of 4 distinct phases: the first gap of mitosis (Gap1: G1), replication of its DNA (synthesis: S), preparation to divide (Gap2: G2), and the mitosis phase (M).^[1]^ The entire cellular process is regulated by many factors, and ubiquitination plays a pivotal role in controlling diverse biological processes. The anaphase-promoting complex/cyclosome (APC/C), a multi-subunit E3 ubiquitin ligase enzyme, has been considered the major driving force governing these cellular processes.^[2]^ APC/C regulates cell cycle progression in both the M and G1 phases and promotes chromosome segregation.^[3]^ APC/C has a complex structure that consists of over 14 different subunits, together with one of the 2 co-activators, CDH1 and cell division cycle 20 homologue (CDC20).^[4]^ CDC20 includes 2 main segments: the C-terminal region containing the WD40 repeats and IR motif and the N-terminal region containing C-box, CRY-box, and KEN-box motifs. Remarkably, both CRY-box and KEN-box are important for CDC20 binding to APC/C as a substrate of CDH1.^[5]^ The CRY-box can lead to timely ubiquitination and destruction of CDC20. Two CDC20 regulators MAD2 and MAD3/ BUBR1 interact with CDC20 through different KEN motifs. Additional regulatory sites are located in the amino- and carboxy-terminal extensions and include the IR tail, C-box, and MAD2-intercating motif, whose function is to bind CDC20 to APC/C (Fig. 1).

**Figure 2.**
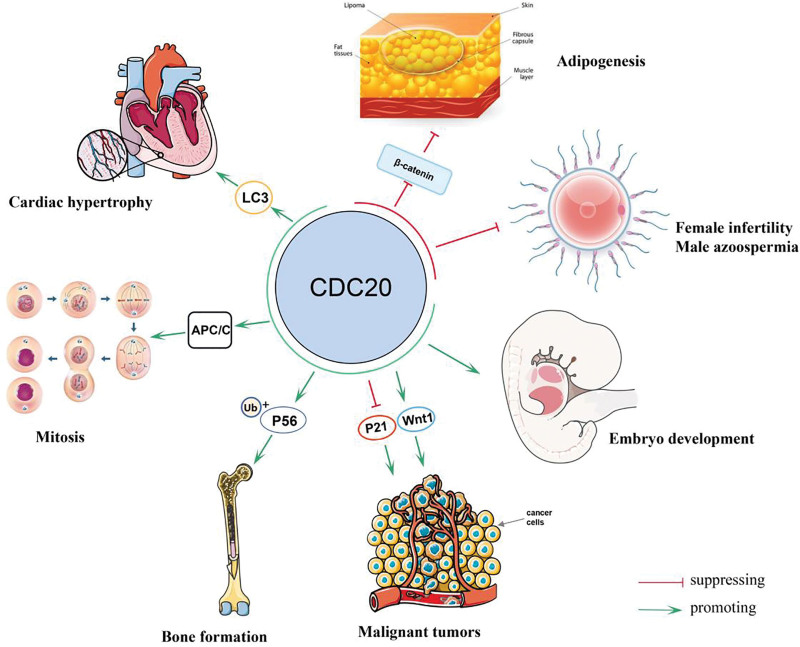
The main human biological processes and many diseases associated with cell division cycle 20 homologue (CDC20).

Accumulating evidence has demonstrated that CDC20 is involved in several biological processes, and many diseases are related to the dysregulation of CDC20. Du et al found that CDC20, as a pivotal regulator, promoted bone formation by governing the ubiquitination and degradation of p65,^[6]^ and CDC20 knockdown enhanced adipogenesis of bone marrow-derived stem cells by modulating β-catenin and might lead to a new therapeutic target for “fatty bone” and osteoporosis.^[7]^ High CDC20 expression in cardiomyocytes aggravates the hypertrophic response by targeting LC3 directly, and which could be a new therapeutic target for patients with hypertrophic heart disease.^[8]^ CDC20 is also spatially expressed in human menstrual cycles and is regulated by estrogen and progesterone, an experiment confirmed that the CDC20 inhibitor, Apcin, can reduce the proliferation and adhesion of human endometrial cells and inhibit embryo implantation in mice.^[9]^ In addition, many studies have demonstrated that mutations in human CDC20 could result in female infertility and male azoospermia.^[10–12]^ Furthermore, CDC20 has been shown to play a crucial role in tumorigenesis, progression, and treatment of human malignant solid tumors (Fig. 2). In the following paragraph, we focus on the expression and role of CDC20 in various solid cancers.

## 2. Role of CDC20 in human malignant solid tumors

Many studies have indicated that mutated CDC20 plays a catalytic role in human tumorigenesis. In addition, the overexpression of CDC20 is associated with progression, treatment resistance, poor prognosis, and clinicopathological features in multiple human cancers.

### 2.1. Aberrant expression of CDC20 in cancer prognosis and tumorigenesis

Many studies have demonstrated that CDC20 is abnormally expressed in various solid cancers, and aberrant expression of CDC20 is associated with tumorigenesis and poor prognosis. For example, CDC20 is upregulated in various types of tumors, including non-small cell lung cancer (NSCLC),^[13]^ hepatocellular carcinoma (HCC),^[14]^ gastric carcinoma (GC),^[15]^ colorectal cancer (CRC),^[16]^ renal clear cell carcinoma,^[17]^ bladder urothelial carcinoma,^[18]^ prostate cancer,^[19]^ epithelial ovarian cancer,^[20]^ and cervical squamous carcinoma^[21]^ relative to matched non-cancerous tissues. Strikingly, CDC20 expression was also significantly higher in intestinal metaplasia, low-grade dysplasia, high-grade dysplasia, and early GC than in normal mucosa, and their expression levels were the highest in high-grade dysplasia, suggesting their roles in the early stage of gastric carcinogenesis.^[22]^ Furthermore, the expression of CDC20 and STK15, identified as CDC20-associated proteins, was enhanced by more than 2-fold in 58% of pancreatic cancer tissues.^[23,24]^ Zhang et al found that CDC20 was overexpressed in smoking-related lung adenocarcinoma, indicating that the levels of CDC20 may correlate with smoking status.^[25]^ A study further demonstrated that hexavalent chromium (Cr [VI]) leads to cancer by altering CDC20 kinetochore localization and reducing the interaction between phosphorylated CDC20 and Mad2.^[26]^ Notably, CDC20 was upregulated in glioblastoma and decreased in low-grade glioma, indicating that additional studies are required to delineate the exact function of CDC20 in gliomas.^[27]^ Recent studies also revealed that speckle-type POZ protein interacts with CDC20 and promotes poly-ubiquitination and subsequent degradation of CDC20 in a degron-dependent manner (tumorigenesis).^[28]^ Therefore, CDC20 acts as an oncogene during tumorigenesis. In addition, multiple studies have indicated that CDC20 is overexpressed significantly along with poor prognosis in astrocytoma,^[29]^ NSCLC,^[13,30]^ HCC,^[14]^ and prostate cancer.^[18]^ Aberrant expression of the GTF2E2/CDC20 signaling pathway is associated with glioblastoma pathogenesis and poor prognosis.^[31]^ Moreover, the expression of CDC20 was associated with the clinicopathological parameters of the tumors mentioned above. High expression of CDC20 was positively correlated with sex, poor tumor differentiation, large tumor size, lymph node invasion, distant metastasis, advanced TNM stage, and cytokine levels in breast cancer,^[32]^ HCC,^[14,33]^ GC,^[22,34]^ CRC,^[16]^ epithelial ovarian cancer.^[20]^ In addition, CDC20 overexpression was associated with advanced age, high grade, non-papillary growth pattern in bladder cancer patients,^[18]^ and Gleason score, vesicle invasion, and biochemical recurrence in prostate cancer,^[19]^ indicating that CDC20 expression is an independent prognostic factor.

These characteristics are consistent with CDC20 as an oncogene. These studies indicate that CDC20 can be used as an independent prognostic factor for human solid cancers (Table 1). Therefore, CDC20 could be a potential prognostic diagnostic marker and independent predictor of biochemical recurrence in human solid cancers.

**Table 1:** The regulation of CDC20 in malignant solid tumors.

Disease	Pathology	Machanism	Function	Reference
Glioblastoma	NA	FoxM1→CDC20 p21CIPI/WAF1	Proliferation	^[35]^
	NA	MIIP CDC20→suppressing Cyclin B1 degradation	Development and progression	^[36]^
	NA	NF2 binding of APC/C and CDC20→restore SAC function	Proliferation	^[37]^
	NA	CDC20→EMT	Temozolomide-resistant	^[38]^
	NA	CDC20 knockdown→accumulation of Bim	Chemo- and radiosensitivity	^[39]^
	NA	CDC20-APC/SOX2 signaling axis	Dispute the stem-like cell	^[40]^
	NA	GTF2E2/CDC20 signaling pathway	Pathogenesis and poor prognosis	^[31]^
Lung cancer	NSCLC	miR-7515/miR-1321 CDC20 EMT	EMT, migration, invasion, proliferation,apoptosis	^[41]^
	NA	P53 CDC20 G2/M phase and colony-forming activity	Suppress Cancer cell growth	^[42]^
Breast cancer	NA	REC8-CDC20 regulating axis	Decreasing proliferation, migration, invasion	^[43]^
	TNBC	A20/TNFAIP3-CDC20-ASP1 axis	Poor prognosis, metastasis, cytokines levels.	^[32]^
	NA	AhR-CDC20 axis	Suppressing mammosphere formation	^[44]^
	NA	CDC20-SMAR1 axis	Progression and apoptosis	^[45]^
Hepatocellular carcinoma	NA	CDC20 altered the subcellular location and distribution of PC-PLC, and degrade PC-PLC by UPP.	Proliferation	^[46]^
	NA	CCT4 inhibits tumor through interacting with CDC20	Tumor growth and pathogenesis	^[31]^
	NA	CDC20-PHD3-HIF1a-VEGF axis	Tumorigenesis	^[47]^
	NA	CDC20 silencing increases E-cadherin, and inhibits N-cadherin, vimentin, Ki-67	Promoting progression by regulating EMT	^[48]^
	P53 mutant	CDC20-Bcl2/Bax pathway	Proliferation and radiosensitivity	^[49]^
Gastric cancer	NA	MYBL2-CDC20-Wnt/β-catenin pathway	Proliferation and apoptosis	^[50]^
	NA	CBX4-CDC20 axis	Proliferation, migration, metastatic	^[51]^
Colorectal cancer	NA	CDC20-conductin/axin2/axil-Wnt/β-catenin pathway	Proliferation, colony formation	^[52]^
Pancreatic cancer	NA	lnRNA SpRY4-IT4 increases CDC20 expression	Proliferation and invasion	^[53]^
	PDAC	CDC20-UBE2S/VHL/HIF-1α/STAT3	Enhancing EMT	^[54]^
Renal carcinoma	NA	CDC20 increases level of securin, cyclin B1, cyclin A	Proliferation and migration	^[55]^
	NA	CYP1B1 can induce CDC20 expression	Proliferation, migration, invasion, inhibiting apoptosis	^[56]^
	NA	miR-182-5-MALAT1 -CDC20 axis	Proliferation, colony formation, apoptosis	^[57]^
Bladder cancer	NA	CDC20 induces radio-resistance by targeting FOXO1 degradation.	Radio-resistance of bladder cancer cells	^[58]^
Prostate cancer	NA	SPOP interact with CDC20	Inhibiting expression of stemness related genes	^[28]^
	NA	Silencing of CDC20 inhibits Wnt/β-catenin pathway	Chemosensitivity, Suppressing castration-resistance	^[50]^
	NA	CDC20 lead to docetaxel resistant in Bim-dependent manner.	Docetaxel resistance	^[59]^
Osteosarcoma	NA	Downregulation of CDC20 enhances Bim and p21 expression.	Chemosensitivity to cisplatin	^[60]^
	NA	NUSAP1 upregulates CDC20, while CDC20 silencing inhibits NUSAP1.	Proliferation and cell cycle	^[61]^

APC/c = anaphase promoting complex/cyclosome, CDC20 = cell division cycle 20 homologue, EMT = epithelial-mesenchymal transition, MIIP = migration and invasion inhibitor protein, NF2 = neurofibromatosis type 2, NSCLC = non-small cell lung cancer, PC-PLC = phosphatidylcholine-specific phosphatase C, SAC = spindle assembly checkpoint, SPOP = speckle-type POZ protein, UPP = ubiquitin proteasome pathway.

### 2.2. Role of CDC20 in altered cell proliferation of cancer cells

Multiple studies have demonstrated that CDC20 plays an important role in promoting and maintaining tumor cell proliferation. Dai et al CDC20 plays a critical role in FoxM1-related cell survival in glioma cells.^[35]^ CDC20 maintains the proliferation and survival of glioma cells through degradation of p21CIPI/WAF1, and CDC20 inhibition induces cell cycle arrest in glioma cells.^[62]^ Furthermore, neurofibromatosis type 2 might restore spindle assembly checkpoint function by impairing the binding of APC/C and CDC20, thereby inhibiting meningioma proliferation.^[37]^ The CDC20-APC/SOX2 signaling axis controls the key biological properties of glioma cells, and targeting this pathway in glioblastoma may dispute the glioblastoma stem-like cell line state.^[40]^ In addition, hyperphosphorylation of CDH1 is a major mechanism that drives attenuated APC/CDC20 activity in the G1 phase of the cell cycle and reduces the viability of glioma cells.^[63]^ Furthermore, CDC20/APC/C/securing-dependent signaling is a key regulator of cell survival, and its disruption promotes premature senescence in normal lung cells.^[64]^ P53 suppressed cancer cell growth by indirectly regulating CDC20 expression, arrests cells at the G2/M phase, and reduces the colony-forming activity of lung cancer cells.^[42]^ Remarkably, CDC20 also promotes cell growth and migration in breast cancer cells, including triple-negative breast cancer cells.^[65]^ CDC20 regulates the growth of breast cancer cells via several pathways. REC8-CDC20 axis: REC8 decreases proliferation, migration, and invasion of breast cancer cells by inhibiting CDC20.^[43]^ AhR-CDC20 axis: AhR activation by 3-methylcholanthrene suppresses mammosphere formation by downregulating CDC20 expression in breast cancer cells.^[44]^ CDC20-SMAR1 axis: The tumor suppressor SMAR1 plays a crucial role in maintaining genomic stability and cell cycle progression, whereas CDC20 promotes proteasomal degradation of SMAR1.^[45]^ In HCC, CDC20 overexpression could also promote cell signal transduction and proliferation by altering the subcellular localization and distribution of phosphatidylcholine-specific phosphatase C (PC-PLC), and causing PC-PLC degradation through the ubiquitin-proteasome pathway.^[46]^ In addition, CDC20-mediated degradation of PHD3 stabilized HIF-1a and promoted HCC tumorigenesis. The depletion of endogenous PHD3 in CDC20 knockdown HCC cells greatly attenuated the decline in HIF-1a protein and restored the secretion of VEGF.^[47]^ The proliferation of p53 mutant HCC cells can also be regulated by CDC20 via the Bcl/Bax pathway.^[49]^ Additionally, Wnt/β-catenin signaling regulates cell proliferation by modulating the cell cycle and is negatively regulated by conductin/ axin2/axil, while conductin is degraded by CDC20, indicating that knockdown of CDC20 blocked Wnt/β-catenin signaling through conductin and attenuated colony formation of CRC cells.^[52]^ In GC, the synergy between CDC20 and MYBL2 induces the proliferation of GC cells, and this effect may have involved the Wnt/β-catenin signaling pathway.^[50]^ Furthermore, CBX4 mainly promotes CDC20 mRNA levels and notably enhances cell proliferation capacity, migration ability, and in vivo metastatic efficacy.^[51]^ In renal cancer, CDC20 promotes proliferation by increasing the protein levels of securin, cyclin B1, and cyclin A.^[55]^ In addition, CYP1B1 may promote renal cell proliferation, migration, and invasion, and inhibit apoptosis by inducing CDC20 expression.^[56]^ miR-182-5 overexpression inhibits cell proliferation by directly targeting MALAT-1, then the downregulation of MALAT-1 leads to a decrease in CDC20.^[57]^ In prostate cancer, L-type amino acid transport protein is expressed at all stages of prostate cancer. Inhibition of L-type amino acid transport suppresses tumor growth and cell cycle progression by downregulating M-phased cell cycle genes, including CDC20 and mTORC1, in prostate cancer.^[66]^ Another study found that COX2 inhibitors suppressed prostate cancer cell proliferation and arrested cell cycle progression by inhibiting CDC20.^[67]^ Moreover, NUSAP1 accelerated osteosarcoma cell proliferation and cell cycle progression by upregulating CDC20, whereas CDC20 silencing inhibited NUSAP1- induced cell proliferation and cell cycle in osteosarcoma cells.^[61]^ CDC20 also promotes the growth of other cancer cells, including pancreatic,^[53,68]^ cervical,^[69,70]^ and ovarian cancers.^[20]^

In general, suppression of CDC20 inhibited cell proliferation by inducing G2/M phase arrest, anti-angiogenesis, and activating various signaling pathways (Table 1). Therefore, CDC20 inhibitors may be an ideal therapeutic strategy to block cancer cell growth.

### 2.3. Impact of CDC20 on apoptosis

Several studies have reported that CDC20 is associated with apoptosis in various solid cancers. A previous study demonstrated that CDC20 inhibition could induce apoptosis in glioma.^[62]^ Hu et al also found that miR-1321 and miR-7515 impede NSCLC cell apoptosis by targeting CDC20.^[41]^ Furthermore, CDC20/APC/C/securing-dependent signaling is a key regulator of cell survival, and its disruption promotes premature senescence in normal lung cells and induces apoptosis in lung cancer.^[64]^ The tumor suppressor SMAR1 plays a crucial role in maintaining genomic stability and apoptosis, whereas CDC20 promotes proteasomal degradation of SMAR1 through K48-linked specific polyubiquitylation, and short hairpin RNA-mediated inactivation of CDC20 leads to significant stabilization of SMAR1.^[45]^ Furthermore, silencing CDC20 expression can activate cell apoptosis by altering the subcellular localization and distribution of PC-PLC and inhibiting PC-PLC degradation, PC-PLC is involved in the cell apoptosis.^[46,71]^ CDC20 also coordinates with MYBL2 to inhibit cell apoptosis through the Wnt/β-catenin signaling pathway.^[50]^ Notably, CYP1B1 may inhibit apoptosis by inducing CDC20 expression.^[56]^ In addition, miR-182-5 overexpression inhibited cell apoptosis by directly targeting MALAT-1, leading to downregulation of CDC20.^[57]^ Other studies have further confirmed that CDC20 could induce apoptosis in prostate cancer^[72]^ and osteosarcoma.^[73]^

Based on the above, the expression level of CDC20 is associated with apoptosis of cancer cells, indicating that inhibiting apoptosis is one of the main mechanisms by which CDC20 promotes cancer cell growth (Table 1). CDC20 regulates apoptosis of cancer cells mainly by interacting with related proteins and the Wnt/β-catenin signaling pathway.

### 2.4. Invasion and metastasis regulatory action of CDC20

CDC20 not only regulates the proliferation of cancer cells, but also promotes invasion and metastasis. For example, migration and invasion inhibitor protein suppressed APC-mediated Cyclin B1 degradation by interacting with CDC20, thereby inhibiting glioma development and progression.^[36]^ CDC20 is negatively regulated by miR-1321 and miR-7515 to suppress NSCLC cell migration and invasion.^[41]^ CDC20 also promotes the migration of triple-negative breast cancer cells, and CDC20 inhibitors would block cancer cell growth and migration.^[65]^ The function of CDC20 in breast cancer is regulated by REC8 and the A20/TNFAIP3-CDC20-ASP1 axis. REC8 decreases proliferation, migration, and invasion of breast cancer cells by inhibiting CDC20.^[43]^ The A20/TNFAIP3-CDC20-CASP1 axis, which includes inflammation-related genes found in TNBC, is associated with poor patient prognosis, cancer metastasis, and cytokines levels.^[32]^ Besides, CBX4 promoted CDC20 mRNA levels and notably enhanced cell migration ability and metastatic efficacy as a positive regulatory factor.^[51]^ The lncRNA SpRY-IT4 also promotes cell invasion by regulating CDC20 in pancreatic cancer cells.^[53]^ Furthermore, CYP1B1 may promote renal cell migration and invasion by inducing CDC20 expression.^[56]^ Suppression of CDC20 slao inhibited migration and resulted in G2/M phase arrest in wilms tumor cells by increasing the expression levels of securin, cyclin B1, and cyclin A.^[55]^ Moreover, several studies have shown that CDC20 knockdown can inhibit the invasion and metastasis of prostate cancer,^[74]^ osteosarcoma,^[75]^ cervical cancer, and ovarian cancer^[69]^ (Table 1).

Furthermore, multiple studies have revealed that CDC20 promotes proliferation, invasion, and metastasis of cancer cells through the CDC20-meidated angiogenesis pathway, indicating that CDC20 can promote angiogenesis in cancer tissues. For example, CDC20 overexpression is involved in TMZ-resistant glioma cells with epithelial-mesenchymal transition (EMT).^[38]^ In addition, miR-1321 and miR-7515 can suppress NSCLC cell EMT by targeting CDC20.^[41]^ CDC20 silencing increased the expression levels of E-cadherin, decreased the expression levels of N-cadherin, vimentin, and Ki-67, and inhibited migration and invasion of HCC cells. Therefore, CDC20 can promote HCC progression by regulating EMT.^[48]^ Meanwhile, CDC20-mediated degradation of PHD3 stabilizes HIF-1a and promotes tumorigenesis in HCC. The depletion of endogenous PHD3 in CDC20 knockdown HCC cells greatly attenuated the decline in HIF-1a protein and restored the secretion of VEGF^[47]^ (Table 1). Notably, co-expression of CDC20 and UBE2S can enhance EMT in pancreatic cancer cells via the VHL/HIF-1α/STAT3 pathway.^[54]^ UBE2S downregulated the expression level of VHL through ubiquitin, forming a ubiquitin complex with VHL and HIF-1α and regulating the transcription factor STAT3. Subsequently, it changes the downstream proteins related to EMT. Moreover, UBE2S could form an important ubiquitin ligase APC/C with ANAPC2/4, whereas CDC20 could regulate APC/C, activating other significant factors in EMT progression, such as E-cadherin by ubiquitin, to affect the invasion and metastasis of pancreatic cancer.

Based on the above, CDC20 can directly promote the invasion and metastasis of solid cancers through various signaling pathways, including the REC8-CDC20, A20/TNFAIP3-CDC20-ASP1, CBX4-CDC20, and VHL/HIF-1α/STAT3 axes. Notably, CDC20 also promotes angiogenesis in tumor tissues by regulating EMT and VEGF expression, which is an important pathway promoting invasion and metastasis.

### 2.5. Modulation of antitumor immunity by CDC20

Multiple studies have demonstrated that CDC20 can regulate the proliferation and progression of cancers through signaling pathways, interactions with related protein factors, and angiogenesis. Recently, some studies have found that the expression level of CDC20 could regulate the antitumor immunity system. For example, the A20/TNFAIP3-CDC20-CASP1 axis, which includes inflammation-related genes found in triple-negative breast cancer, is associated with cytokines levels.^[32]^ Besides, CDC20 expression was significantly correlated with immune infiltration in HCC. There was a loss of association between CDC20 and Th1/Th17 cytokines, monocytes, CD4pos, and CD8pos T cells, as well as exhausted T cells.^[76]^ The positive correlation between the CDC20 gene and inhibitory checkpoint genes PD-1, CTLA4, and TIM-3 indicates that the CDC20 gene is linked with weakened antitumor immunity in HCC.^[76]^ Furthermore, CDC20 expression is positively correlated with the degree of infiltration of B cells, neutrophils, macrophages, and myeloid dendritic cells.^[77]^ In addition, CDC20 has been linked to the tumor mutation burden, immune checkpoint molecules, tumor microenvironment, and immunological infiltration.^[17]^ Therefore, CDC20 plays a critical role in the antitumor immunity system, such as the expression of cytokines, activation of immune cells, expression of immune checkpoint molecules, microenvironment (Table 1). CDC20 could be a novel biomarker for predicting immunotherapy outcomes and immune landscapes in patients and may be an immune-associated therapeutic target in cancers.

### 2.6. CDC20 and antitumor therapy resistance

CDC20 not only regulates cancer cell proliferation and progression but also promotes resistance to chemotherapy and radiotherapy. Mao et al found that CDC20 knockdown regulated chemosensitivity and radiosensitivity in glioblastoma through the accumulation of the pro-apoptotic protein Bim.^[39]^ Lfarsi et al also found that high CDC20 expression was correlated with a poor response to endocrine treatment in ER + breast cancer patients treated with hormonal therapy.^[78]^ In addition, CDC20 was related to chemotherapy-resistant ER + breast cancer cells; MGMT inhibition led to CDC20 inhibition and increased the response to temozolomide and alkylator-based chemotherapy.^[79]^ Therefore, CDC20 could act as a potential predictive biomarker of poor response to endocrine therapy and chemotherapy in ER + breast cancer. CDC20 can also regulate the radiosensitivity of P53 mutant HCC cells through the Bcl-2/Bax pathway.^[49]^ In addition, knockdown of CDC20 enhanced the cytotoxicity of paclitaxel and increased the effect of gamma irradiation against pancreatic carcinoma cells.^[80]^ CDC20 could also induce the radioresistance of bladder cancer cells by targeting FOXO1 degradation, indicating that the inactivation of CDC20 might be a potential strategy to overcome radioresistance in bladder cancer.^[58]^ Recently, several studies have shown that CDC20 is associated with chemosensitivity in prostate cancer. For example, Zhang et al demonstrated that knockdown of CDC20 inhibited chemoresistance in prostate cancer.^[74]^ Silencing of CDC20 can enhance chemosensitivity to docetaxel in metastatic castration-resistant prostate cancer by inhibiting Wnt/β-catenin signaling.^[81]^ CDC20 overexpression can also facilitate docetaxel resistance in prostate cancer cells in a Bim-dependent manner.^[59]^ Moreover, downregulation of CDC20 enhanced chemosensitivity to cisplatin in osteosarcoma cells, while Bim and p21 were upregulated in osteosarcoma cells following apcin treatment.^[60]^ Id1 conferred 5-fluoroutacil chemoresistance in esophageal cancer cell through E2F1-denpendent induction of IGF2 and thymidylate synthase, a critical target of anticancer drugs especially 5-fluoroutacil.^[82]^ While Id1 can increase E2F1 expression by binding competitively to CDC20, suppressing CDC20 can lead to E2F1 degradation, reversing the Id1-E2F1-IGF2 regulatory axis to enhance the chemosensitivity of esophageal cancer cells.^[82]^

Therefore, high expression of CDC20 is critical for radiotherapy, chemotherapy, and endocrine resistance (Table 2). Suppressing CDC20 could be a promising strategy to overcome resistance to antitumor therapy in various cancers.

**Table 2:** CDC20 and antitumor therapy resistance.

Disease	Resistance	Machanism	Reference
Giloblastoma	Chemo- and radiotherapy	CDC20 knockdown accumulate the pro-apoptotic, Bim.	^[39]^
Breast cancer (ER+)	Endocrine treatment	NA (clinical trail)	^[78]^
	Chemotherapy	CDC20 inhibitor induce p21 and c-PARP expression.	^[79]^
HCC (P53 mutant)	Radiosensitivity	CDC20 regulate the radiosensitivity through Bcl-2/Bax pathway.	^[49]^
Pancreatic carcinoma	Paclitaxel Gamma-irradiation	Suppression of CDC20 induced accumulation of the cells in the G2/M-phase of the cell cycle.	^[80]^
Bladder cancer	Radiotherapy	CDC20 induced the radio-resistance by targeting FOXO1 degradation	^[58]^
Prostate cancer	Chemosensitivity	(1) Silencing CDC20 enhance chemosensitvity by inhibition of Wnt/β- catenin signaling. (2) CDC20 overexpression facilitate the docetaxel resistant by inhibiting Bim expression.	^[59,81]^
Osteosarcoma	Chemosensitivity	Apcin upregulate the Bim and p21.	^[60]^
Esophageal cancer	5-fluoroutacil	CDC20 can increase E2F1 expression by binding Id1.	^[82]^

CDC20 = cell division cycle 20 homologue, HCC = hepatocellular carcinoma.

## 3. The prospect of CDC20-target inhibition

Given the important oncogenic role of CDC20 in tumorigenesis, targeting CDC20 may interfere with mitosis, thereby inhibiting cancer proliferation. Therefore, we have summarized several CDC20 inhibitors with therapeutic effectiveness in suppressing tumor progression (Table 3). Some inhibitors have previously been reported to be effective in the treatment of cancers, including CFM-4, TAME/pro-TAME, Apcin, Withaferin A, NAHA, Ganodermanontriol and Mycophyto complex, Genistein, PRoTACs (CP5V), and BCHHD7c.^[91,92]^ Recently, some novel compounds that target CDC20 have been reported. For example, some CDC20 inhibitors have been successfully used in the treatment of glioma, such as rottlerin isolated from the medicinal plant Mallotus philippinensis, and MLN8237, a Food and Drug Administration-approved AURKA inhibitor, selectively killed temozolomide-resistant primary glioma cells and prolonged the survival of patients (Table 3).^[83,84]^ Liu et al further showed that ZINC000004098930 was chosen as the ideal natural ligand to target and inhibit CDC20, which may greatly contribute to TNBC-targeted treatment.^[94]^ Remarkably, several drugs targeting CDC20, including NaBt (histone deacetylase inhibitor), Withaferin A, and 1-L-MT (a canonical IDO inhibitor), have been developed to kill cancer cells by delaying mitotic exit followed by inducing chromosome instability.^[85,86,93]^ Bhuniya et al designed new apcin-based inhibitors by replacing the pyrimidine group with substituted thiazole-containing groups, which can improve antitumor activity.^[87]^ M2I-1 (MAD2 inhibitor-1) has been shown to disrupt the CDC20-MAD2 interaction, and consequently, the assembly of the mitotic checkpoint complex and increase the sensitivity of cancer cells to antimitotic drugs via MCL-1s.^[88]^ Moreover, a triterpene mixture extracted from the mushroom Poria cocos, polyporenic acid C, can downregulate the expression of CDC20 in some cancer cells and inhibit cancer metastasis.^[89]^ Zhang et al have identified a compound as “331” which upregulate miR-494 and downregulated CDC20 to induce cell death in glioma cells but not in astrocytes.^[90]^

**Table 3:** The list of all compounds targeting CDC20 activity.

Compound	Information	Target and function	Reference
Rottlerin	It also celled mallotoxin, is isolated from plant Mallotus phillippinensis.	Rottlerin exert its tumor suppressive function by inhibiting CDC20 expression.	^[83]^
MLN8237	It also named Alisertib and is benzazepine-fused pyrimidinering compound that inhibit Aurora.	AURKA is a core member of CDC20-M, MLN8237 is a AURKA inhibitor and kill temozolomide-resistant primary glioma cells.	^[84]^
NaBt	Sodium butyrate, a short chain fatty acid, is a histonedeacetylases (HDACs) inhibitor.	A HDAC inhibitor totally downregulated p55CDC/CDC20 transcription and expression.	^[85]^
1-L-MT	A canonical IDO inhibitor, Indoleamine 2,3-dioxygenase 1 (IDO1), known as IDO, catabolizes tryptophan through kynurenine pathway.	Preventing cancer by inducing CDC20	^[86]^
A new Apcin-based CDC20 inhibitors	Developing a new apcin-based inhibitors by eliminating a controlled substance, chloral hydrate, required for synthesis. It improved the antitumor activities of the inhibitors by replacing the pyrimidine group with substituted thiazole-containing groups.	A new series of CDC20 inhibitors base on apcin by eliminating a controlled substance, chloral hydrate, required for synthesis.	^[87]^
M2I-1	The first small molecule inhibitor targeting the binding of Mad2 to CDC20.	A MAD2 inhibitor, suppresses the CDC20-MAD2 interaction and enhance the drug-sensitivities.	^[88]^
Triterpene mixture	A triterpene mixture extracted from the mushroom Poria cocos, polyporenic acid C.	Downregulate the expression of CDC20 in cancer cells, the specific mechanism is till under study.	^[89]^
Compound 331	A hybrid Fe chelator derived from di-2-pyridylketone-4,4,-dimethyl-3- thiosemicarbazone.	Upregulates miR-494 and downregulated CDC20.	^[90]^
CFM-4	CARP-1 functional mimetic 4, CARP-1 is a peri-nuclear phosphoprotein that regulates cell growth and apoptosis.	Down-regulates Cdc20 in breast cancer cells and induces apoptosis.	^[91]^
TAME	Tosyl-l-arginine methyl ester is an inhibitor of cyclin proteolysis in mitotic Xenopus egg extract. It is a mimetic of the IR motif.	Reduces CDC20 association with the APC and subsequently inhibits APC activity.	^[92]^
Pro-TAME	A TAME pro-drug can be processed by intracellular esterases to yield the active form of TAME.	Disrupting the APC-Cdc20 interaction and then reduces APC activation.	^[92]^
Apcin	It is an APC inhibitor binding CDC20 and prevent substrate recognition, thereby leading to competitively inhibition of the ubiquitination of CDC20 substrates.	Occupies the D-box-binding pocket on the side of the WD40 domain and blocks substrate-induced Cdc20 loading onto the APC.	^[92]^
Withaferin A	A bioactive component from Withania somnifera.	Enhances degradation of Cdc20, blocks SAC function, leading to mitotic delay.	^[93]^
NAHA	A N-alkylated amino acid-derived sulfonamide hydroxamate.	Inhibits the expression of Cdc20 in breast cancer cells, retards cell proliferation and colony formation.	^[92]^
Ganodermanontriol and Mycophyto complex	GDNT is a ganoderma alcohol from medicinal mushroom. MC is a novel medicinal mushroom blend.	Down-regulates Cdc20 expression and inhibits cell proliferation and invasion in breast cancer cells.	^[92]^
Genistein	A phytoestrogenic isoflavonid as a protein tyrosine kinase inhibitor.	Regulation of Cdc20 in various human cancers to exert its anti-tumor activity.	^[92]^
PROTACs (CP5V)	Apcin-A-PEG5-VHL Ligand 1, a prteolysis targeting chimera.	Degrades Cdc20 and overcomes cell division slippage	^[92]^
BCHHD 7c	The 6-brominated coumarin hydrazide-hydrazone derivative 7c.	Inhibits Cdc20 expression in drug resistant pancreatic cancer cells.	^[92]^

CDC20 = cell division cycle 20 homologue, SAC = spindle assembly checkpoint.

However, given the fluctuations in CDC20 expression during cell cycle progression, the adverse side effects of CDC20 inhibitors must be considered. CDC20 is also a crucial factor in cell cycle regulation in normal cells. Thus, targeting CDC20 may cause defects in the cell cycle of normal tissues. Therefore, it is necessary to fully understand the modulation of CDC20 expression and activity during the cell cycle.

## 4. Conclusion and future perspective

**Figure 3.**
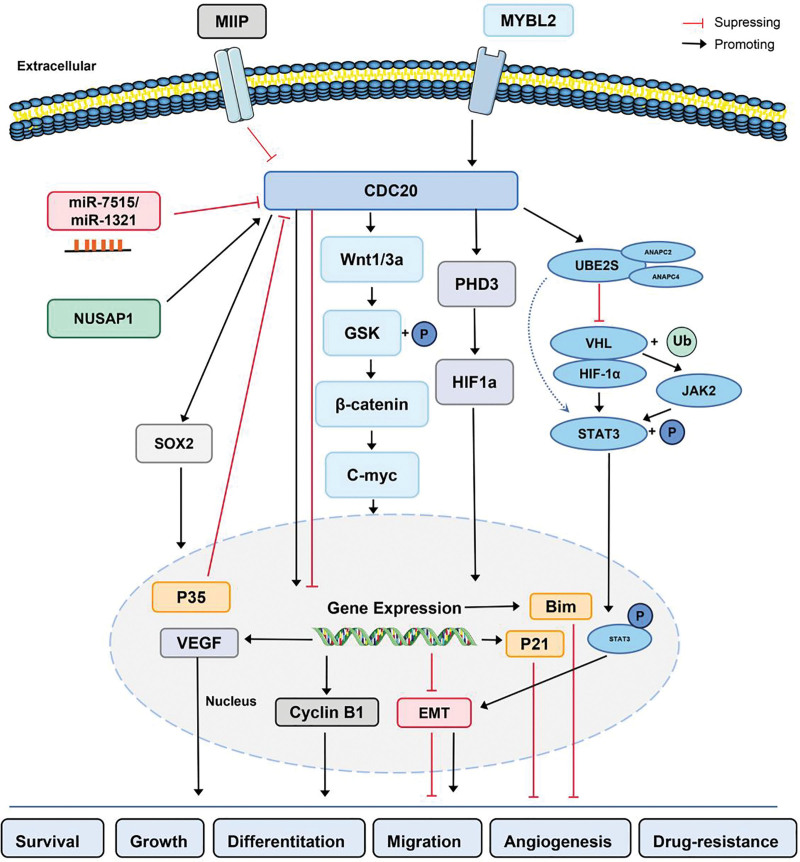
The main signaling pathway of cell division cycle 20 homologue (CDC20) in cancers.

In summary, CDC20 acts as an oncogene through various pathways, including the promotion of EMT, suppression of apoptosis, inhibition of immunological infiltration, and signaling pathways. In addition, we briefly summarize the pathway for CDC20-degradation of its major downstream targets as well as the identified CDC20 upstream regulators, and the effects of CDC20 on solid tumors (Fig. 3). Lastly, we introduced the newest CDC20 inhibitors that can decrease cancer metastasis and kill cancer cells. Therefore, CDC20 plays an important role in the development and progression of human cancers, and the development of specific CDC20 inhibitors could provide a valuable breakthrough in cancer treatment and intervention. To determine the role of CDC20 in future studies, 2 important issues need to be addressed. CDC20 is involved in several biological activities; therefore, we need to develop a CDC20 inhibitor with greater specificity and efficacy for cancer treatment to avoid severe adverse effects, due to those current CDC20 inhibitors have toxic and nonspecific nature. However, further investigation is required to explore the mechanisms underlying CDC20-mediated tumorigenesis. Furthermore, the expression, role, and regulatory mechanism of CDC20 in some cancers, such as intrahepatic cholangiocarcinoma, remains unclear and requires further exploration. To fully explore the function of CDC20 in tumors, CDC20 conditional knockout or knock-in mouse models are necessary to better understand the physiological role of Cdc20 in various human cancer settings.

## Acknowledgments

The authors are grateful to Dr Yinglin Yuan, School of Medicine, University of Electronic Science and Technology of China; for her great help and support during the progress of the work.

## Author contributions

**Conceptualization:** Guohui Xu.

**Data curation:** Feng Xian, Caixia Zhao, Guohui Xu.

**Formal analysis:** Feng Xian, Chun Huang, Guohui Xu, Jun Bie.

**Funding acquisition:** Feng Xian, Guohui Xu.

**Investigation:** Feng Xian, Chun Huang.

**Methodology:** Feng Xian, Caixia Zhao, Chun Huang, Jun Bie.

**Resources:** Feng Xian.

**Software:** Feng Xian, Caixia Zhao.

**Supervision:** Feng Xian.

**Validation:** Feng Xian.

**Visualization:** Feng Xian.

**Writing – original draft:** Feng Xian, Guohui Xu.**Writing – review & editing:** Guohui Xu, Jun Bie.
